# Mental Maladies; a Treatise on Insanity

**Published:** 1845-10

**Authors:** 


Art. III.—*Mental Maladies; a Treatise on Insanity.* By E. Esquirol. Translated from the French, with Additions, by E. K. Hunt, m.d.—*Philadelphia,* 1845. 8vo, pp. 496.

M. Esquirol's treatise has been so long acknowledged as a standard work on insanity, that it is surprising it has never been translated before. We think, therefore, that the readers and speakers of the English language, on both sides the Atlantic, are much obliged to Dr. Hunt for the trouble he has taken to accommodate such of them as do not understand the original, with the present version of it. "All that portion of the treatise, relating properly to insanity, has been published [in the translation] entire; the remainder, referring, for the most part, to the statistics and hygiene of establishments for the insane, together with the medico-legal relations of the subject, have been omitted." The translator has introduced here and there, a little original matter, relating chiefly to the opinions and practice adopted in the United States. So far, we can recommend the work to our readers. We are sorry, however, to be obliged to add that as a literary composition, it is extremely defective,—bad as a translation, worse as an English book; and consequently discreditable to Dr. Hunt, both as a French and English scholar. And we are sorry to say that many of the American translators which we have seen of late, are obnoxious to the same charge. We tell our good friends on the other side of the Atlantic, to look to this betimes; if they are not more careful about their style of writing, they will, in the end, come to differ as much from their forefathers in language, as they do in civil polity; and this, we presume, they have no ambition to do.

We put down, at random, a few samples of Dr. Hunt's manifold delinquencies:—

"Unsuitable marriages; those contracted by *parents;* and above all those, &c." (p. 42.)

"The same may be said of the great lords of France who were almost all of them *parents,* &c.," (p. 49,)—meaning relatives or relations.

"He *pretends* to be blindly obeyed," (p. 74,)—meaning *insists* on being blindly obeyed.

"Jesus Christ has a *fine figure,*" (p. 100,)—meaning a *beautiful face.*

"His teeth were *poor,*" (p. 114.)

"Render a *well* person epileptic," (p. 168.)

"Engages in the *exercise* of *the chase,*" (p. 176,)—meaning *goes out shooting.*

"Since *the turns* have been abundant," (p. 192,)—meaning *menstruation.*

"She finds herself feeling unwell," (p. 263,)—elle se trouve mal.

"Falls a victim to *a reflected resolution,*" (p. 274.)

"G., *a proprietary,* leaves seven children," (p. 277.)

"Lively moral affections," (p. 470,)—meaning just the opposite.

He talks of the *savage* of Avignon,—instead of the *wild man,*—indeed always translates "sauvage" *savage,* where it means *wild.* (p. 486.) *Cannot,* he invariably makes "knows not how to," &c. *Huit jours* and *quinze jours,* he renders "eight days" and "fifteen days," instead of *week* and *fortnight.*—Talks of "provoking" the bowels.—Speaks of a *falling out* with her companion, and being *at one again.*—Translates always "embrasser" *embrace,* instead of kiss.

